# Supplementary material for: A Simple and User-Friendly Method for High-Quality Preparation of Pollen Grains for Scanning Electron Microscopy (SEM)
Source: Plants (Basel). 2024 Aug 1;13(15):2140. doi: 10.3390/plants13152140 (PMC11314231; doi:10.3390/plants13152140)
Supplement: Supplementary file 1 [file plants-13-02140-s001.zip › figS3.pdf]

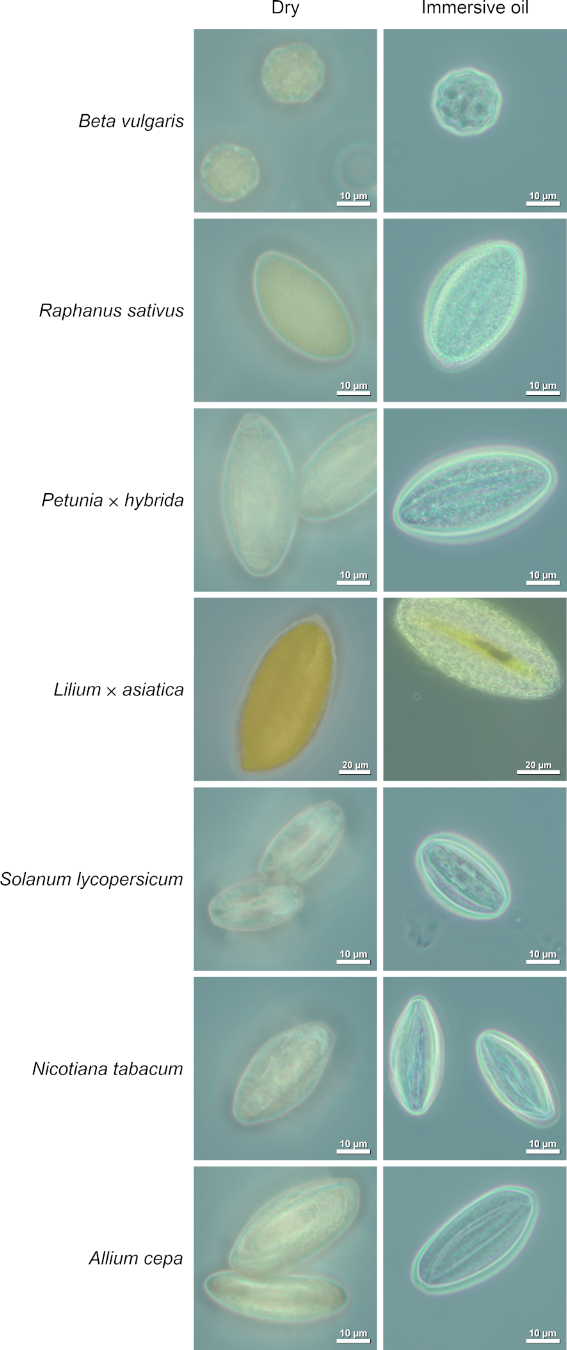
Dry
Immersive oil
Beta vulgaris
Raphanus sativus
Petunia × hybrida
Lilium × asiatica
Solanum lycopersicum
Nicotiana tabacum
Allium cepa
10 µm
20 µm
