# Supplementary figures and images for: A Simple and User-Friendly Method for High-Quality Preparation of Pollen Grains for Scanning Electron Microscopy (SEM)
Source: Plants (Basel). 2024 Aug 1;13(15):2140. doi: 10.3390/plants13152140 (PMC11314231; doi:10.3390/plants13152140)

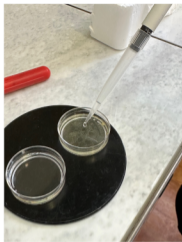

**A**

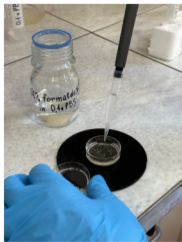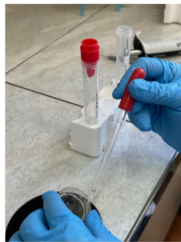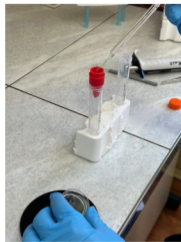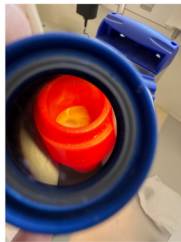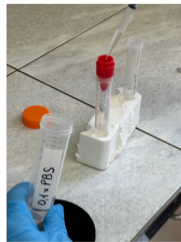

**C**

**B**

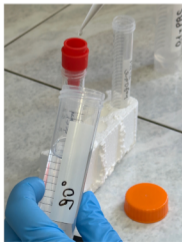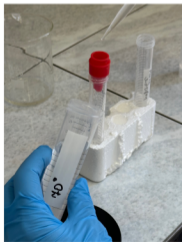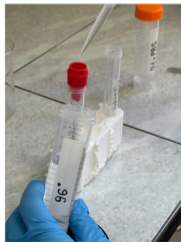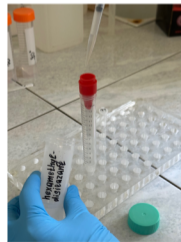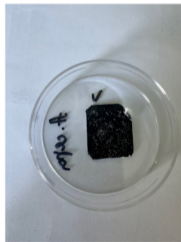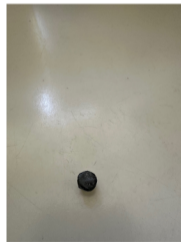

**D**

**E**

**F**

**G**

Supplement: Supplementary file 1 [file plants-13-02140-s001.zip › figS1.pdf]

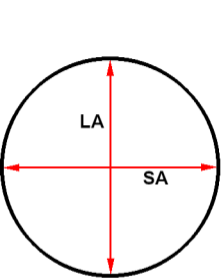

**a**

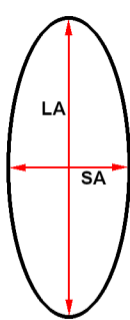

**b**

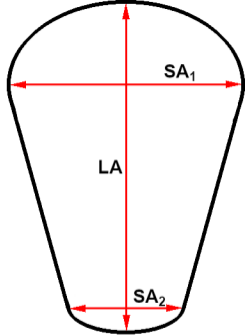

**c**

Supplement: Supplementary file 1 [file plants-13-02140-s001.zip › figS2.pdf]

Dry

Immersive oil

*Zea mays*

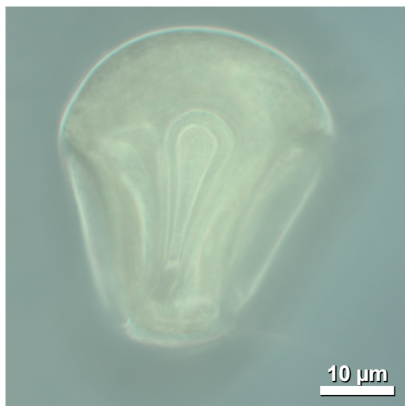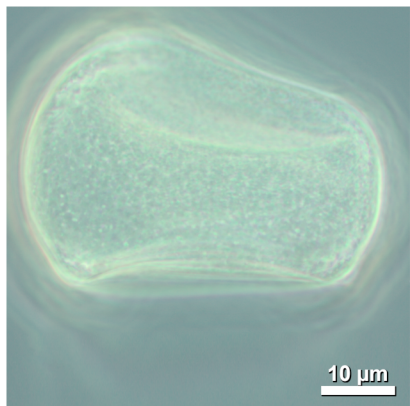

*Secale cereale*

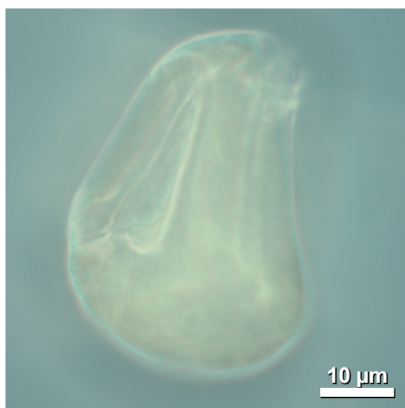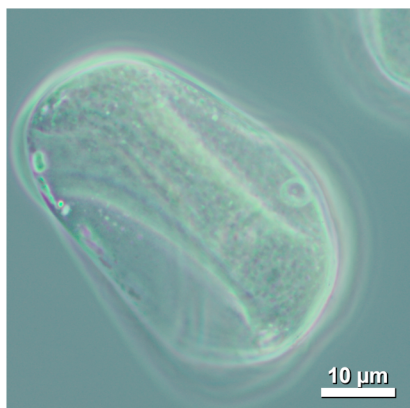

*Triticum aestivum*

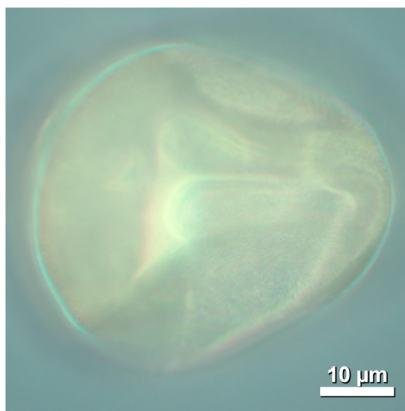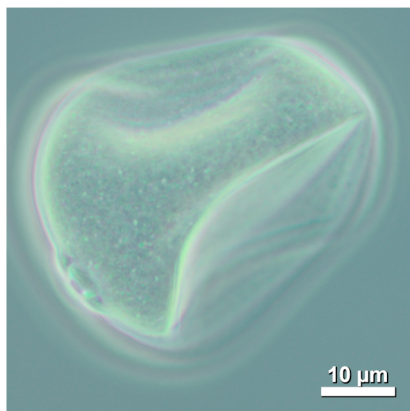

Supplement: Supplementary file 1 [file plants-13-02140-s001.zip › figS4.pdf]
